# FuNP (Fusion of Neuroimaging Preprocessing) Pipelines: A Fully Automated Preprocessing Software for Functional Magnetic Resonance Imaging

**DOI:** 10.3389/fninf.2019.00005

**Published:** 2019-02-11

**Authors:** Bo-yong Park, Kyoungseob Byeon, Hyunjin Park

**Affiliations:** ^1^Department of Electrical and Computer Engineering, Sungkyunkwan University, Suwon, South Korea; ^2^Center for Neuroscience Imaging Research, Institute for Basic Science, Suwon, South Korea; ^3^School of Electronic and Electrical Engineering, Sungkyunkwan University, Suwon, South Korea

**Keywords:** functional magnetic resonance imaging, data preprocessing, volume- and surface-based preprocessing, fully automated software, fusion of existing software

## Abstract

The preprocessing of functional magnetic resonance imaging (fMRI) data is necessary to remove unwanted artifacts and transform the data into a standard format. There are several neuroimaging data processing tools that are widely used, such as SPM, AFNI, FSL, FreeSurfer, Workbench, and fMRIPrep. Different data preprocessing pipelines yield differing results, which might reduce the reproducibility of neuroimaging studies. Here, we developed a preprocessing pipeline for T1-weighted structural MRI and fMRI data by combining components of well-known software packages to fully incorporate recent developments in MRI preprocessing into a single coherent software package. The developed software, called FuNP (Fusion of Neuroimaging Preprocessing) pipelines, is fully automatic and provides both volume- and surface-based preprocessing pipelines with a user-friendly graphical interface. The reliability of the software was assessed by comparing resting-state networks (RSNs) obtained using FuNP with pre-defined RSNs using open research data (*n* = 90). The obtained RSNs were well-matched with the pre-defined RSNs, suggesting that the pipelines in FuNP are reliable. In addition, image quality metrics (IQMs) were calculated from the results of three different software packages (i.e., FuNP, FSL, and fMRIPrep) to compare the quality of the preprocessed data. We found that our FuNP outperformed other software in terms of temporal characteristics and artifacts removal. We validated our pipeline with independent local data (*n* = 28) in terms of IQMs. The IQMs of our local data were similar to those obtained from the open research data. The codes for FuNP are available online to help researchers.

## Introduction

Functional magnetic resonance imaging (fMRI) is a useful tool for exploring brain functions non-invasively. The preprocessing of raw fMRI data is an essential step before performing further analyses because of the following reasons. First, fMRI measures spontaneous fluctuations of blood oxygen-level dependent (BOLD) signals that are related to neuronal activities. However, BOLD signals contain non-neuronal contributions, such as head motion, physiological contributions, tissues outside the scope of interest, and MRI-induced artifacts, as well as neuronal signals (Murphy et al., 2013; Bright and Murphy, 2015; Caballero-Gaudes and Reynolds, 2017). The non-neuronal components in BOLD signals complicate the interpretation of fMRI signals. Secondly, the quality of fMRI data largely depends on the image acquisition parameters used. Different MRI data might have a different range of intensity values, matrix sizes, and orientations depending on the acquisition parameters used. Thus, preprocessing steps for fMRI data are required to handle these issues.

In previous studies, researchers have developed freely available open-source neuroimaging data preprocessing tools, such as statistical parametric mapping (SPM)^1^, analysis of functional neuroimages (AFNI) (Cox, 1996), FMRIB software library (FSL) (Jenkinson et al., 2012), FreeSurfer (Fischl, 2012), Workbench (Marcus et al., 2013), and fMRIPrep (Esteban et al., 2019). These are widely used software tools, but each one of them employs a different strategy for data preprocessing. SPM and FSL provide fully automated graphical user interface (GUI)-based preprocessing pipelines and are suitable for volume data. FreeSurfer is suitable for surface data and provides a fully automated command line-based pipeline. AFNI and Workbench process both volume and surface data, but they do not provide a fully automated pipeline in a user-friendly interface. Users need to rearrange different functions in these disparate software tools if they seek to implement automatic data preprocessing. Different data preprocessing strategies across different software packages might yield differing results, which might reduce the reproducibility of the neuroimaging studies. The fMRIPrep is a recent development incorporating many of the state-of-the-art MRI preprocessing steps.

There are many steps in a given preprocessing pipeline, including field inhomogeneity correction, motion correction, registration, and segmentation steps. Many of these steps are standardized, but some of them are still being actively developed and refined to better preprocess fMRI data. For example, many researchers argue that cortical signals are better handled via surface-based approaches, while sub-cortical signals are better handled via volume-based approaches (Glasser et al., 2013, 2016a,b). Data-driven approaches, such as independent component analysis (ICA), to identify unwanted signals are being increasingly adopted (Salimi-Khorshidi et al., 2014; Pruim et al., 2015a,b). Time-series volume data with large head movements are sometimes removed based on frame-wise displacement (FD) (Power et al., 2012; Damaraju et al., 2014; Yeo et al., 2015). To the best of our knowledge, no single software package has all the recent developments fully incorporated. Thus, neuroimaging researchers are forced to integrate different components of various software packages if they seek to adopt all the recent developments in fMRI preprocessing.

Here, we propose a novel software for fMRI data preprocessing, named FuNP (Fusion of Neuroimaging Processing) pipelines, a wrapper software that combines components of existing software tools (i.e., AFNI, FSL, FreeSurfer, and Workbench) to fully incorporate recent developments in MRI preprocessing. Such wrapper software might be of practical impact for researchers with limited data processing background. Our software consists of preprocessing steps for structural (T1-weighted MRI) and functional (fMRI) data. We assessed the reliability of our software by comparing resting-state networks (RSNs) obtained using FuNP with pre-defined RSNs because it is difficult to obtain the ground truth of the preprocessing results. In addition, the quality of the preprocessed data was assessed using the image quality metrics (IQMs) proposed in the previous paper (Esteban et al., 2017). The major advantages of our software are as follows. FuNP can handle both volume- and surface-based preprocessing. The software is fully automated and has a user-friendly GUI.

## Materials and Methods

FuNP provides two different types of fMRI preprocessing steps: (1) volume-based and (2) surface-based preprocessing pipelines. Both preprocessing pipelines include steps to process structural (T1-weighted MRI) and functional (fMRI) data. In the volume-based pipeline, data are preprocessed in 3D volume space. Volume-based analysis has been widely adopted in many neuroimaging studies. In the surface-based pipeline, data are preprocessed both in volume and surface spaces. The surface-based pipeline operates in 2D surface space but requires intermediate outcomes from volume analyses. In this pipeline, the cortical regions are represented as a 2D surface, while the sub-cortical regions are represented as a 3D volume. This mixing of surface and volume spaces is a recent development, and some researchers have claimed that it can improve the sensitivity of neuroimaging studies (Glasser et al., 2013, 2016a,b). Our software provides flexibility to perform each of the preprocessing steps. Users can select “Yes” or “No” options for every step in our software to selectively perform the steps as required. Furthermore, users can select user specified parameters for each step. For example, the degrees of freedom (DOF) and cost functions for registration could be specified in the GUI. Details of each preprocessing steps can be found in following sections.

### Volume-Based T1-Weighted MRI Data Preprocessing

**FIGURE 1:**
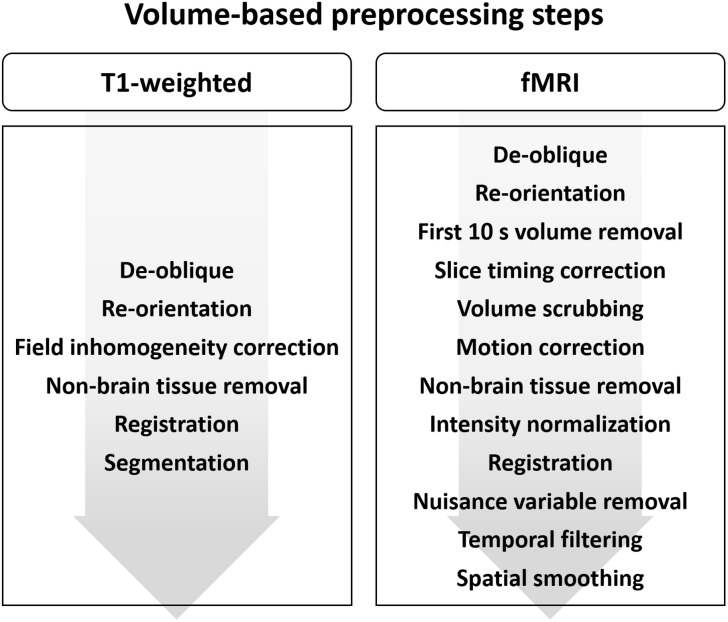
Diagram of the preprocessing steps for volume-based **(A)** T1-weighted structural MRI and **(B)** fMRI data.

The volume-based preprocessing steps for T1-weighted structural data are presented in Figure 1.

#### De-Oblique

**FIGURE 2:**
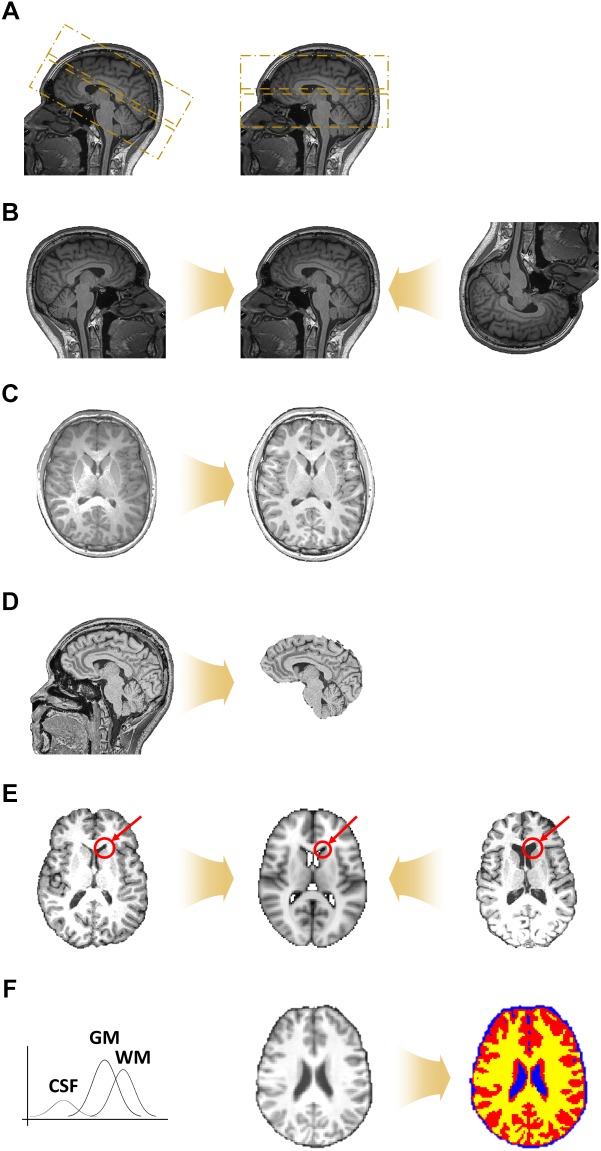
Preprocessing steps for volume-based T1-weighted structural MRI data. **(A)** De-oblique step. Example images of (left) tilted and (right) non-tilted data are shown. **(B)** Matched data with different orientations to the same orientation. **(C)** Magnetic field inhomogeneity correction. **(D)** Non-brain tissue removal. **(E)** Registration onto the standard space. **(F)** Segmentation of brain tissues into gray matter (GM; red), white matter (WM; yellow), and cerebrospinal fluid (CSF) (blue).

During data acquisition, the scan angle is sometimes tilted from the horizontal line (i.e., between the anterior and posterior commissure) to cover the whole brain and to avoid MRI-induced artifacts caused by air and water in the eyes and nose (Figure 2A). Such a tilted scan is referred to as an oblique scan. Oblique scans enable us to acquire data with less noise, but can make the registration between two different images more difficult. Thus, a de-oblique process needs to be performed. De-oblique is performed using the “3drefit” function in AFNI (Cox, 1996).

#### Re-orientation

The orientation of data depends on the settings of the data acquisition process (Figure 2B). Differences in orientation might lead to mis-registration, and thus all data should be matched to have the same orientation. Orientation is specified with a three-element vector: (1) left or right, (2) anterior or posterior, and (3) superior or inferior. For example, if the right, posterior, and inferior directions are chosen, the orientation of the data is called RPI. Orientation can be defined in any way but should be the same for all data. Re-orientation is performed using the “3dresample” function in AFNI (Cox, 1996).

#### Magnetic Field Inhomogeneity Correction

The brain consists of different tissues, namely gray matter (GM), white matter (WM), and cerebrospinal fluid (CSF). The magnetic field within the scanner should be constant but, in reality, it decreases when it encounters brain tissue, and the decreasing rate differs across different tissue types (Cheng et al., 2017). This phenomenon is referred to as magnetic field inhomogeneity. These differences in the magnitude of the magnetic field cause abnormally bright and dark areas, which make it difficult to detect tissue boundaries (Figure 2C). Thus, magnetic field inhomogeneity correction should be performed before the non-brain tissue removal and tissue segmentation steps. Magnetic field inhomogeneity correction is performed using the “3dUnifize” function in AFNI by making intensity values in WM more homogeneous (Cox, 1996).

#### Non-brain Tissue Removal

The region of interest (ROI) of neuroimaging studies lies within the brain. Non-brain tissues, such as those of the skull, neck, eyes, nose, and mouth, are thus not important (Figure 2D). The non-brain tissue removal step is performed by considering the gradient of the intensity values across different types of tissues. Non-brain tissue removal is performed using the “3dSkullStrip” function in AFNI (Cox, 1996).

#### Registration

Registration is the process of aligning images from different geometric spaces to a common space (Figure 2E). There are three main components of registration. First, a spatial geometric transformation needs to be specified. The 3D transformation parameters are translation, rotation, scaling, and shearing in the x-, y-, and z-directions. Rigid-body transformation consists of six DOF, involving three translations and three rotations, while affine transformation consists of 12 DOFs involving three scaling and three shearing factors in addition to the rigid-body parameters, which we adopt in FuNP. Secondly, a cost function that measures the goodness of alignment has to be specified. In FuNP, users can select either the correlation ratio or mutual information as the cost function. The correlation ratio is useful when registering two images of the same modality, while mutual information is useful for images from different modalities. Finally, an interpolation method has to be specified. In FuNP, the trilinear interpolation technique is used. Registration is performed using the “flirt” function in FSL (Jenkinson et al., 2012).

#### Segmentation

It has been shown that the fluctuations of time series in GM are associated with neuronal signals, while those in WM and CSF are related to artifacts (Salimi-Khorshidi et al., 2014). Thus, distinguishing between GM, WM, and CSF tissues is important for extracting signals of interest. The Gaussian mixture model distribution is used for discriminating between GM, WM, and CSF tissues (Figure 2F). Segmentation is performed using the “fast” function in FSL (Jenkinson et al., 2012).

### Volume-Based fMRI Data Preprocessing

The volume-based preprocessing steps for fMRI data are presented in Figure 1.

#### Removal of the First N Volumes

**FIGURE 3:**
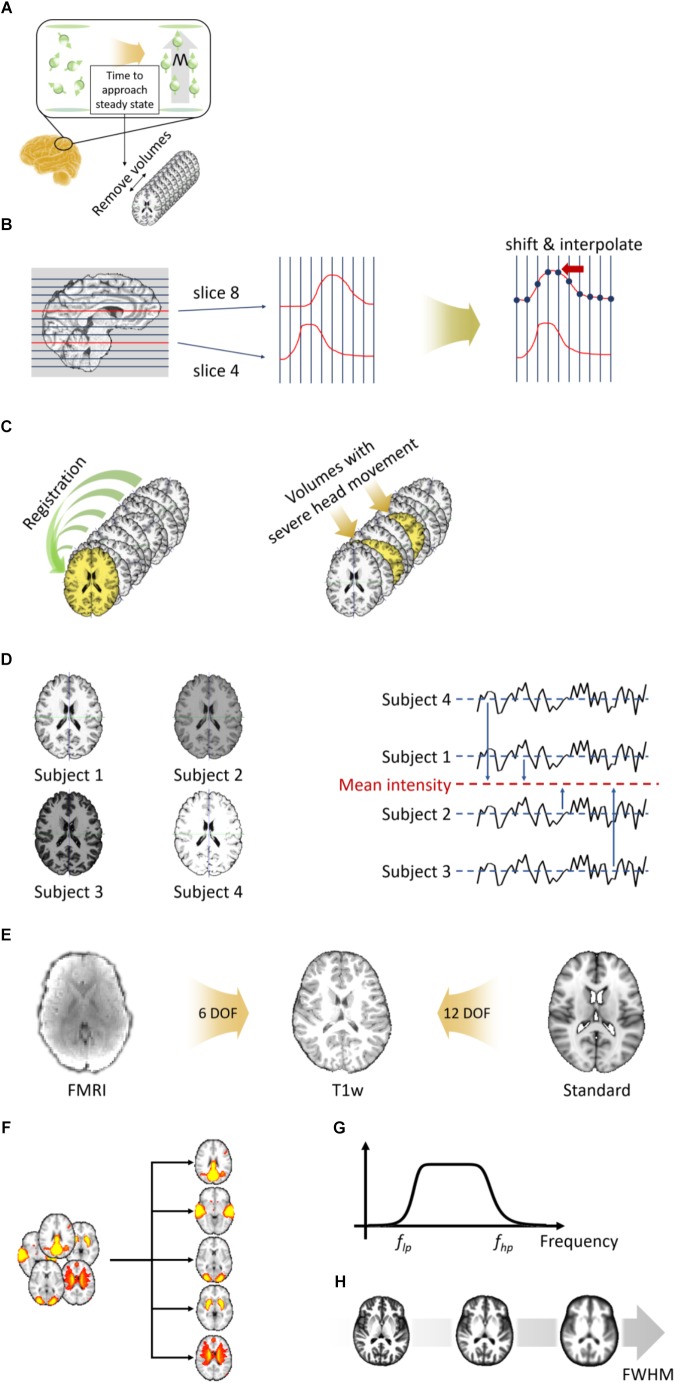
Preprocessing steps for volume-based fMRI data. **(A)** Removal of the first few volumes. **(B)** Slice timing correction. **(C)** Head motion correction (left) and volume scrubbing (right). **(D)** Intensity normalization. **(E)** Two-stage registration. **(F)** Nuisance variable removal via ICA-FIX. **(G)** Temporal filtering. **(H)** Spatial smoothing.

The de-oblique and re-orientation steps are first performed on fMRI data as described in Section “Volume-Based T1-Weighted MRI Data Preprocessing.” The next step is to remove the first few volumes. When a magnetic field is applied to the brain, hydrogen molecules are aligned in the direction of the magnetic field. It takes from 5 to 6 s for these molecules to approach to the steady state, and thus the volumes acquired during the first few seconds (typically 10 s) have to be removed (Figure 3A) (Bright and Murphy, 2015; Bijsterbosch et al., 2017). This process is performed using the “fslroi” function in FSL (Jenkinson et al., 2012).

#### Slice Timing Correction

Slice timing correction is performed to correct the time differences at which each slice was acquired. For example, as shown in Figure 3B, the time of the signal evoked at slice 8 is shifted toward that of slice 4 to match the starting time. The shifted signal is then interpolated. Because the slice timing correction approach uses interpolation, it causes a temporal smoothing effect, which might cause loss of information. Thus, this step is not recommended if the repetition time (TR) of the fMRI data is short (<1 s) (Bijsterbosch et al., 2017). Slice timing correction is performed using the “slicetimer” function in FSL (Jenkinson et al., 2012).

### Motion Correction and Volume Scrubbing

Participants are instructed not to move their heads during an MRI scan. However, there are always unavoidable head movements, and thus the data becomes corrupted with motion-related artifacts. Thus, head motion correction should be performed on all fMRI data. Motion correction is performed by registering all volumes to a reference volume via a rigid-body transformation (Figure 3C). The reference volume can be any volume, but typically the first or middle volume of the whole data is selected. The next step is to remove volumes with severe head motion. This approach is referred to as volume scrubbing (Power et al., 2012). As the rigid-body transformation is used, three translation parameters (with their units in millimeters) and three rotation parameters (with their units in degrees) are calculated. These six motion parameters are used to calculate FD, which measures the degree of head motion (Power et al., 2012). Volumes whose FD exceed 0.5 mm are considered to have severe head motions and are thus removed. Volumes with severe head motion are detected using the “fsl_motion_outliers” function and motion correction is performed using the “mcflirt” function in FSL (Jenkinson et al., 2012).

#### Field Map Correction

After head motion correction, field inhomogeneity correction can be performed. This step requires the collection of a dedicated field map. However, many neuroimaging studies, especially older ones, did not collect field map data and thus we make this step optional. This was intentional so that our software could be applied to many existing neuroimaging studies. If a certain study has a field map-corrected EPI data (e.g., computed using FSL), the user can supply this data as an optional input to our software and the program will proceed with the rest of the pipeline using the field map-corrected data.

#### Intensity Normalization

Because MRI data does not have a specific unit, different MRI data might have different ranges of intensity values. Intensity normalization is performed to standardize the range of intensity values across all 4D volumes with a specific value (Figure 3D). In FuNP, a value of 10,000 is used. Intensity normalization is performed using the “fslmaths” function in FSL (Jenkinson et al., 2012).

#### Registration

Unlike T1-weighted structural MRI data, the resolution of fMRI data is lower and has lower inter-tissue contrast. Thus, it is difficult to directly register fMRI data to the standard space. In FuNP, two-stage registration is adopted (Figure 3E) (Jenkinson et al., 2012; Glasser et al., 2013). Low-resolution fMRI data is registered onto high-resolution preprocessed T1-weighted structural MRI data of the same subject via a rigid-body transformation. The T1-weighted structural MRI data is then registered onto the standard space via an affine transformation. The two transformation matrices are concatenated and then applied to the fMRI data to register them onto the standard space. Registration is performed using the “flirt” function in FSL (Jenkinson et al., 2012).

#### Nuisance Variable Removal

The fMRI data contains both signal and noise components. The noise components include head motion, WM, CSF, cardiac pulsations, and arterial and large vein-related contributions. The noise components can be removed via ICA-FIX (Figure 3F) (Salimi-Khorshidi et al., 2014). ICA is a method for decomposing fMRI signals into a set of spatially independent components (ICs) (Beckmann and Smith, 2004; Beckmann et al., 2005). The computed ICs are further classified into signal and noise components considering their temporal and spatial features (Salimi-Khorshidi et al., 2014). This classification procedure is performed using a hierarchical classification model described in a previous study and it successfully removed artifacts (Salimi-Khorshidi et al., 2014). There are automatic methods to classify ICs, but their performance can be unreliable at times (Kelly et al., 2010; Griffanti et al., 2017). Thus, a manual approach to classify ICs is recommended. The following three major aspects have to be considered to distinguish between signal and noise components. First, spatial maps of signal components largely overlap with GM, while those of noise components overlap with WM, CSF, and blood vessels (Kelly et al., 2010; Griffanti et al., 2017). Secondly, the time series of signal components are relatively stable without sudden spikes (Kelly et al., 2010; Griffanti et al., 2017). Components with sudden isolated spikes in their time series are often classified as head motion-related artifacts. Finally, the frequency spectrum of signal components usually occupies the low-frequency range (<0.1 Hz), while that of noise components occupies a variable band (Kelly et al., 2010; Griffanti et al., 2017). Once the noise components are defined, they are regressed out from the original fMRI data. Nuisance variable removal is performed using the “fix” function in FSL (Jenkinson et al., 2012). The FuNP uses the pre-trained datasets that were trained using different image acquisition settings provided by the FSL team^2^. Thus, the users do not need to manually train their data but choose from one of the several choices that best suits the input data.

#### Temporal Filtering

The signals of interest of fMRI data are known to exist in the low-frequency range (<0.1 Hz) (Biswal et al., 1995; Boubela et al., 2013). However, extremely low-frequency signals (<0.01 Hz) are considered as slow drifts (i.e., non-neuronal signals) (Biswal et al., 1995; Boubela et al., 2013). Thus, band-pass filtering with a frequency range between 0.009 and 0.08 Hz is widely used to capture the signals of interest (Figure 3G). The cut-off frequencies are slightly different across studies, but filtering ranges of 0.008–0.09 Hz and 0.01–0.1 Hz are typically considered (Biswal et al., 1995; Margulies et al., 2010; Yeo et al., 2011; Boubela et al., 2013). In FuNP, users can select either low-pass, high-pass, or band-pass filters with user-set cut-off frequencies. Temporal filtering is performed using the “3dFourier” function in AFNI (Cox, 1996).

#### Spatial Smoothing

Spatial smoothing is achieved by calculating the weighted average over neighboring voxels using a Gaussian kernel and yields blurred data (Figure 3H). The full width at half maximum (FWHM) of the kernel is usually set as two times the voxel size (Worsley and Friston, 1995; Mikl et al., 2008). Spatial smoothing offers the advantage of reducing noise, but it also can lower the intensity of the signal. Therefore, researchers need to proceed with caution when applying spatial smoothing. Spatial smoothing is performed using the “3dmerge” function in AFNI (Cox, 1996).

### Surface-Based T1-Weighted MRI Data Preprocessing

**FIGURE 4:**
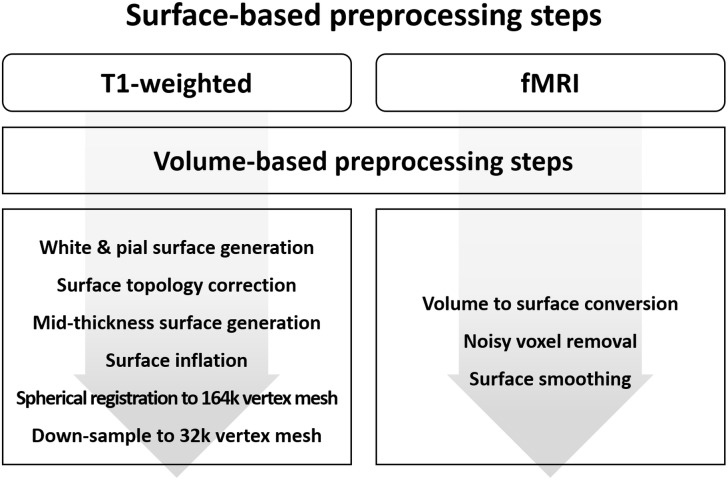
Diagram of the preprocessing steps for surface-based **(A)** T1-weighted structural MRI and **(B)** fMRI data.

The surface-based preprocessing steps of MRI data contain both volume and surface processing steps. This is because the surface processing steps require output from the volume processing steps. The required volume processing steps are largely the same as those described in the previous sections. The surface-based preprocessing steps for T1-weighted structural data is presented in Figure 4. Initial surface-based preprocessing is performed using the “recon-all” function in FreeSurfer (Fischl, 2012). For volume processing, magnetic field inhomogeneity correction, non-brain tissue removal, intensity normalization, segmentation, and registration are performed. For surface processing, white and pial surfaces are generated. The white surface is located between WM and GM, while the pial surface is located between GM and CSF. These white and pial surfaces are generated by following the boundaries between different tissues. The surfaces are then inflated to spheres, and spherical registration between the T1-weighted structural data and the standard space is performed. The surfaces constructed using FreeSurfer are adjusted to obtain accurate surfaces using Workbench as follows (Marcus et al., 2013). The T1-weighted volume data preprocessed using FreeSurfer are registered onto the standard space via an affine transformation. Afterward, the transformation matrix is applied to the white and pial surfaces to register them onto the standard space. These surfaces are then averaged to generate a mid-thickness surface, which is in turn used to generate an inflated surface. The spherical surface is finally registered onto a 164k vertex mesh and then down-sampled to a 32k vertex mesh.

### Surface-Based fMRI Data Preprocessing

The surface-based preprocessing steps for fMRI data also contain volume and surface processing steps. The volume preprocessing steps are the same as those described in Section “Volume-Based fMRI Data Preprocessing” except for spatial smoothing (Figure 4). Spatial smoothing is only performed to subcortical areas and not to cortical areas. The surface-based preprocessing steps are performed using Workbench and FSL (Jenkinson et al., 2012; Marcus et al., 2013). The preprocessed fMRI cortical volume data are converted into surface data to define vertices within the GM ribbon using a cortical ribbon-constrained algorithm (Glasser et al., 2013). Voxels with high variation in their time series (>0.5 standard deviation [SD] of the mean variation of other voxels in a 5-mm neighborhood) are not converted into a surface because they usually contain large blood vessels (Glasser et al., 2013). Surface smoothing on the cortical areas is applied with a FWHM value of twice the voxel size (Worsley and Friston, 1995; Mikl et al., 2008).

### Experiments

The reliability of the developed software was assessed by constructing RSNs using preprocessed resting-state fMRI (rs-fMRI) data obtained from the Human Connectome Project (HCP) database (Van Essen et al., 2013). We hypothesized that if the data were preprocessed properly, the obtained RSNs should be consistent with existing known RSNs. To compare the quality of the preprocessed data from FuNP and other software, we compared our results with those from volume-based preprocessing pipeline using FSL (Jenkinson et al., 2012) and fMRIPrep (Esteban et al., 2019). The IQMs proposed in the previous paper (Esteban et al., 2017) were calculated from the preprocessed data of three different software packages (i.e., FuNP, FSL, and fMRIPrep).

#### Participants and Imaging Data

The data used in this study came from two sources. The first dataset was obtained from the HCP database. We used all the data in the Q3 release version which had both T1-weighted and rs-fMRI data, which led to 90 healthy subjects (58% female) (Van Essen et al., 2013). The mean age was 28.74 with an SD of 3.42. The Institutional Review Board (IRB) of Sungkyunkwan University approved this retrospective study, and it was performed in full accordance with local IRB guidelines. All participants provided written informed consent. All imaging data were obtained using a Siemens Skyra 3T scanner at Washington University. The imaging parameters of the T1-weighted structural data were as follows: TR = 2,400 ms; echo time (TE) = 2.14 ms; field of view (FOV) = 224 mm × 224 mm; voxel size = 0.7 mm isotropic; and number of slices = 256. The imaging parameters for rs-fMRI were as follows: TR = 720 ms; TE = 33.1 ms; FOV = 208 mm × 180 mm; voxel size = 2 mm isotropic; number of slices = 72; and number of volumes = 1,200.

An additional 28 T1-weighted structural MRI and rs-fMRI data of healthy subjects (100% female) were recruited from Sungkyunkwan University to assess the reproducibility of our software. The mean age was 23 with an SD of 2.09. All subjects provided written informed consent according to the procedures approved by the IRB of Sungkyunkwan University. The imaging data were obtained using a Siemens Skyra 3T scanner at Sungkyunkwan University. The imaging parameters of the T1-weighted structural data were as follows: TR = 2,400 ms; TE = 2.34 ms; FOV = 224 mm × 224 mm; voxel size = 0.7 mm isotropic; and number of slices = 224. The imaging parameters for rs-fMRI were as follows: TR = 1,000 ms; TE = 39.8 ms; FOV = 224 mm × 224 mm; voxel size = 2 mm isotropic; number of slices = 72; and number of volumes = 360.

#### RSN Construction

RSNs were defined via an ICA approach (Minka, 2000; Himberg and Hyvärinen, 2003; Beckmann and Smith, 2004; Beckmann et al., 2005; Calhoun et al., 2009). Volume-based preprocessed rs-fMRI data were temporally concatenated across all subjects and fed into the “melodic” function in FSL (Beckmann and Smith, 2004; Beckmann et al., 2005; Jenkinson et al., 2012). The number of dimensions was automatically determined via principal component analysis (PCA) (Minka, 2000; Beckmann and Smith, 2004; Beckmann et al., 2005). The generated volume-based ICs (VICs) were classified as signal and noise components via visual inspection (Kelly et al., 2010; Griffanti et al., 2017). The signal VICs were compared with known RSNs via cross-correlation to see whether the generated VICs were similar to the pre-defined RSNs (Smith et al., 2009).

Surface-based preprocessed rs-fMRI data were handled using the ICASSO approach on the temporally concatenated voxel-wise time series across all subjects^3^ (Himberg and Hyvärinen, 2003). This was done because FSL cannot perform ICA on surface-based preprocessed rs-fMRI data. The generated surface-based ICs (SICs) were visually compared with the known RSNs because there are no openly available RSN data in surface format.

#### Comparison With Other Software

We compared the results of FuNP with those from volume-based preprocessing pipeline using FSL (Jenkinson et al., 2012) and fMRIPrep (Esteban et al., 2019). The comparison was limited to volume-based approaches as FSL did not provide surface-based results. The preprocessing steps of FSL were as follows: the first 10 s volumes were removed and head motion was corrected. The non-brain tissue was removed using the temporally averaged fMRI data. The noise reduction process was performed using a non-linear filtering. The intensity normalization, high-pass filtering, and spatial smoothing were applied. The fMRI data were registered onto the T1-weighted structural data and then consequently onto the MNI standard space. The preprocessing steps of fMRIPrep were as follows: a reference volume and its skull removed data were generated. Head motion and susceptibility distortions were corrected. The distortion corrected data were registered onto the T1-weighted structural data and then consequently onto the MNI standard space. The nuisance variables including head motion, physiological regressors, and global signals of WM, CSF, and the whole brain were removed. The ICA-based Automatic Removal Of Motion Artifacts (ICA-AROMA) was performed to remove the head motion-related artifacts (Pruim et al., 2015b). High-pass filtering was applied and then volumetric resampling configured with Lanczos interpolation was applied to minimize the smoothing effect. The quality of the preprocessed data was assessed using the IQMs proposed in the previous paper (Esteban et al., 2017). The IQMs that assess the temporal information were (1) SD of DVARS (D means temporal derivative of time series, VARS means root mean square variance over voxels) that measures the rate of BOLD signal changes and (2) temporal signal-to-noise ratio (tSNR). The IQMs that assess the artifacts were (1) mean FD that measures the amount of displacement of the head motion, (2) percentage of the volumes with large head motion over the whole volumes, (3) ghost-to-signal ratio (GSR) in x- and (4) y-directions, (5) AFNI’s outlier ratio (AOR) that calculates number of outliers across the time series, and (6) AFNI’s quality index (AQI) that represents mean quality index by measuring whether the intensity values of each volume are not very different from norm of the whole volumes. We also compared the computational performances among the three software packages. The computational performances were measured using running time and peak memory usage over a subset of HCP data (*n* = 10). The software packages were allowed access to a single-thread CPU resource. The size of the input data (format of .nii.gz) was 1.67 GB on average. Our computation node was equipped with Intel Xeon CPU E5-2637 v3 and 256 GB of memory.

## Results

### Developed Software

**FIGURE 5:**
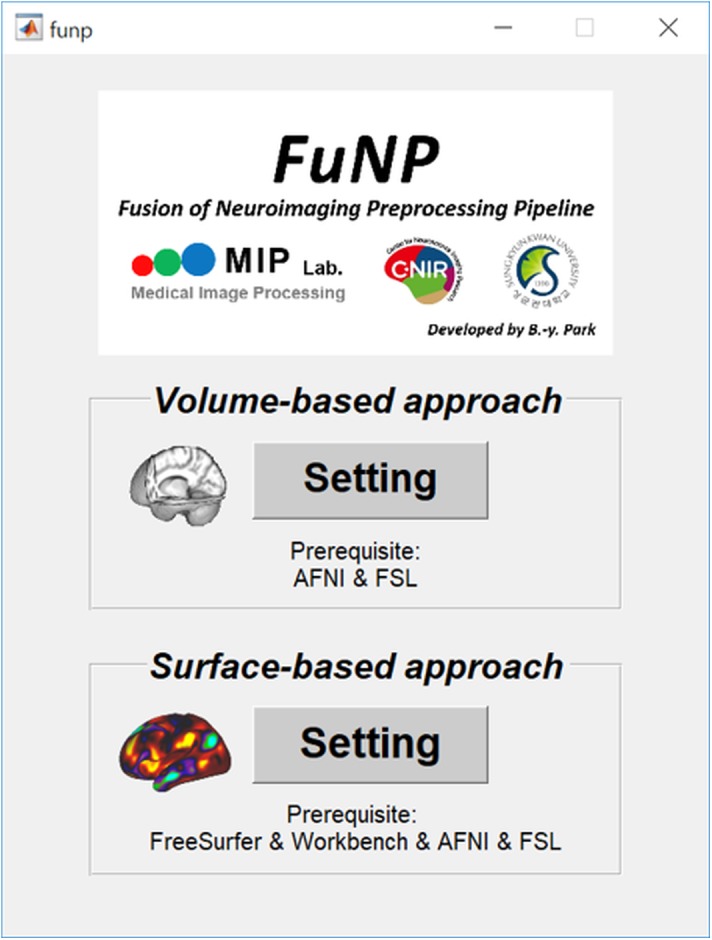
Screenshot of the developed software, called FuNP.

We developed a novel data preprocessing software, called FuNP (Figure 5), for T1-weighted structural MRI and fMRI data. FuNP consists of volume- and surface-based preprocessing approaches. The volume-based approach requires AFNI and FSL (Cox, 1996; Jenkinson et al., 2012), and the surface-based approach requires AFNI, FSL, FreeSurfer, and Workbench (Cox, 1996; Fischl, 2012; Jenkinson et al., 2012; Marcus et al., 2013). Each approach performs the preprocessing of T1-weighted structural MRI and fMRI data separately. Our software, FuNP, is available at in GitLab^4^.

### Reliability of the Software

**FIGURE 6:**
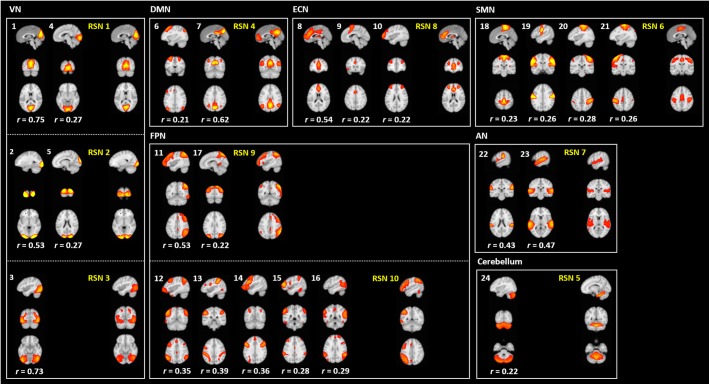
Generated VICs using the HCP data (labeled in a white font) along with pre-defined RSNs (labeled in a yellow font) (Smith et al., 2009). The cross-correlation values of the spatial maps between the generated VICs and RSNs are presented.

To assess the reliability of the output of FuNP, we constructed volume- and surface-based RSNs using the HCP rs-fMRI data preprocessed by FuNP. A total of 29 VICs were automatically generated and classified as 24 signals and 5 noise components (Figure 6). VICs 1–5 were the visual network (VN), consisting of the superior-, middle-, and inferior-occipital gyri, cuneus, and the lingual gyrus. VICs 6 and 7 were the default mode network (DMN), consisting of the superior- and middle-frontal gyri, the medial orbitofrontal gyrus, and the posterior cingulate cortex. VICs 8–10 were the executive control network (ECN), consisting of the middle- and medial-orbitofrontal gyri and anterior cingulate cortex. VICs 11–17 were the frontoparietal network (FPN), consisting of the middle- and inferior-orbitofrontal gyri and the superior- and inferior-parietal lobule. VICs 18–21 were the sensorimotor network (SMN), consisting of the paracentral lobule and the postcentral gyrus. VICs 22 and 23 were the auditory network (AN), consisting of Heschl’s gyrus, the superior temporal gyrus, and the supramarginal gyrus. VIC 24 was the cerebellum. These 24 functionally interpretable VICs were compared with pre-defined RSNs by computing cross-correlation (Smith et al., 2009). The mean cross-correlation value was 0.38, with an SD of 0.17. The results obtained with FuNP showed high similarities between the generated VICs and the pre-defined RSNs, indicating that the data were properly preprocessed.

**FIGURE 7:**
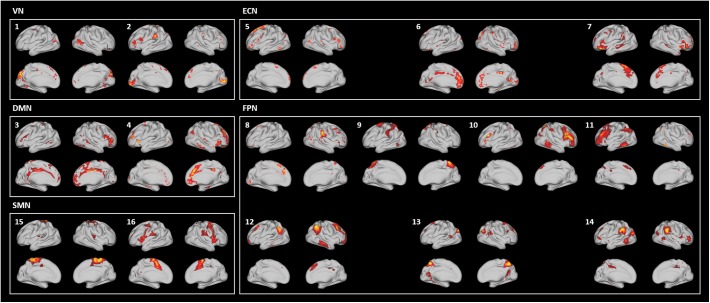
Generated SICs using the HCP data matched with known RSNs.

In addition to the VICs, 20 SICs were generated and classified as 16 signal and 4 noise components (Figure 7). SICs 1 and 2 were the VN, consisting of the primary visual cortex (V1), the early visual cortices (V2 and V3), and the extrastriate visual cortices [V3A, V6, V6A, middle temporal (MT), and middle superior temporal (MST)]. SICs 3 and 4 were the DMN, consisting of the dorsolateral prefrontal cortex, the medial- and inferior-frontal cortices, the anterior- and posterior-cingulate cortices, and the insula. SICs 5–7 were the ECN, consisting of the dorsolateral prefrontal cortex, the medial orbitofrontal cortex, the inferior frontal cortex, and the anterior cingulate cortex. SICs 8–14 were the FPN, consisting of the dorsolateral prefrontal cortex, the medial- and inferior-frontal cortices, the superior- and inferior-parietal lobules, and the paracentral lobule. SICs 15 and 16 were the SMN, consisting of the somatosensory and motor cortices, the premotor cortex, and the paracentral lobule. Regions of the AN were partly included in the SICs of the FPN (SICs 10, 11, 12, and 14). The SICs showed similar patterns to those of known RSNs, suggesting that the preprocessing pipeline was reliable.

### Comparison With Other Software

**FIGURE 8:**
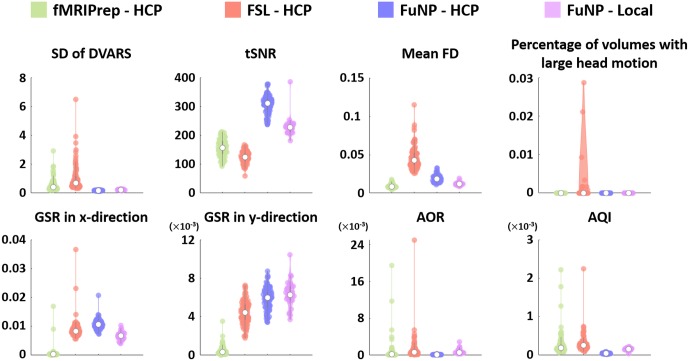
The IQMs of the volume-based preprocessed rs-fMRI data using different software packages. The values were plotted using violin plots. The white circle denotes the median value. The AOR and AQI were very small but the results of some software packages had high variability.

The quality of the volume-based preprocessed rs-fMRI data from FuNP, FSL, and fMRIPrep was assessed using the IQMs (Esteban et al., 2017). We found that FuNP yielded lower SD of DVARS compared to other software. The mean FD and percentage of volumes with large head motion of FuNP were comparable to fMRIPrep and lower than FSL. The results suggest the head motion-related artifacts were better removed using FuNP (Figure 8). The tSNR and GSR showed higher values in FuNP compared to other software indicating the processed data using FuNP were robust to noise (Figure 8). In addition, AOR and AQI showed smaller values for FuNP suggesting there was a smaller number of outliers compared to other software (Figure 8). Taken together, our FuNP outperformed other software in terms of temporal characteristics and artifacts removal.

### Comparison of Computational Resources

We measured the computational performances among the three software packages using running time and peak memory usage. On average, the running time was approximately 3 h for FuNP, 11 h for fMRIPrep, and 11 h and 30 min for FSL (Table 1). Possible reasons behind the longer computation time for fMRIPrep could be different head motion correction and registration methods compared to ours. The FuNP took 6 min, while fMRIPrep took 86 min on average for the motion correction (Table 1). The FuNP was faster (12 min) than fMRIprep (4 h and 52 min) for the registration procedure on average (Table 1). During the 4D data registration, fMRIPrep splits the 4D data into 3D volumes and performs registration onto the reference space. The results of the registration were stored on a disk for all 3D volumes and later concatenated to form the 4D registered data. The operations involve many disk input/output operations and thus could be slow. Our FuNP performs the entire procedure all within the memory and thus does involve fewer disk input/output operations than fMRIPrep. This could lead to faster computation for FuNP. For both fMRIPrep and FSL, the longer computation time might be due to the use of different noise removal strategies. The FuNP was faster (1 h and 29 min) than the two approaches (fMRIPrep; 9 h and 25 min, FSL; 11 h) (Table 1). The fMRIPrep performs nuisance variable removal by calculating various kinds of confounds of mean global signal, mean tissue class signal, PCA-based noise areas defined by anatomy or temporal variance, FD, DVARS, six head motion parameters, respectively (Esteban et al., 2019). In addition, ICA-AROMA for head motion-related artifact removal is performed if the option is set. In contrast, FuNP only uses ICA-FIX that showed good performance of noise removal (Salimi-Khorshidi et al., 2014). In addition, the use of complex non-linear noise filtering algorithm, smallest univalue segment assimilating nucleus (SUSAN), across the whole time series might affect the computation time (Smith and Brady, 1997). In contrast, FuNP only does temporal filtering using a conventional Fourier transform and spatial smoothing for noise removal. Although simple approaches were adopted in FuNP, it exhibited lower outlier ratio compared to other software packages (Figure 8). In terms of peak memory usage, FuNP used 12.5 GB on average, fMRIPrep used 33.1 GB, and FSL used 9.5 GB. Note that the peak memory usage was dependent on the size of the input data. In summary, the running time for the whole preprocessing was fastest when the FuNP was adopted compared to fMRIPrep and FSL.

**Table 1:** Computation time of each preprocessing step for three software packages.

	fMRIPrep	FSL	FuNP
First N volumes removal	N/A	7.4 m(0.5 m)	7.4 m(0.5 m)
Slice timing correction	N/A	N/A	N/A
Volume scrubbing	N/A	N/A	26.8 m(0.4 m)
Motion correction	1 h 26.6 m(6.3 m)	2.2 m(0.4 m)	6.2 m(0.8 m)
Non-brain tissue removal	6.4 m(5.0 m)	4.2 m(0.4 m)	4.6 m(0.5 m)
Intensity normalization	N/A	1.2 m(0.4 m)	1.2 m(0.4 m)
Registration	4 h 52.0 m(1 h 3.2 m)	1.1 m(0.3 m)	12.0 m(2.0 m)
Nuisance variable removal	9 h 25.0 m(1 h 45.2 m)	11 h 0.2 m(1 h 8.8 m)	1 h 29.2 m(10.5 m)
Temporal filtering	1.1 m(0.3 m)	3.2 m(0.4 m)	3.2 m(0.4 m)
Spatial smoothing	N/A	1.8 m(0.4 m)	1.8 m(0.4 m)
Total	11 h 6.6 m(12.9 m)	11 h 35.0 m(52.6 m)	2 h 55.0 m(11.2 m)

*Means and SDs are reported. SD values are reported in parentheses. h, hour; m, minute; N/A, not available.*

### Reproducibility of the Software

To assess the reproducibility of our software, FuNP, we performed additional data preprocessing using local data (*n* = 28). The quality of the results was assessed using IQMs. Figure 8 shows that the IQMs of the preprocessed local and HCP data using FuNP were similar. In addition to the IQMs, we performed volume- and surface-based ICA and found results (from local data) that were similar to the main results (HCP data) (Figure 6, 7 and Supplementary Figures S1, S2). Taken together, we believe our pipeline could yield reproducible results based on the analyses of two independent data sets.

## Discussion

In this study, we developed a preprocessing pipeline for T1-weighted structural MRI and fMRI data by combining components of well-known software packages, namely AFNI, FSL, FreeSurfer, and Workbench, to fully incorporate recent developments in MRI preprocessing into a single software package (Cox, 1996; Fischl, 2012; Jenkinson et al., 2012; Marcus et al., 2013). The developed software, FuNP, is not the first wrapper software that incorporates recent developments in MRI preprocessing. The fMRIPrep is a notable software package that incorporates many of the state-of-the-art MRI preprocessing steps from existing software tools of AFNI, FSL, FreeSurfer, and ANTs (Esteban et al., 2019). They reported that the pipeline is robust to the acquisition parameters of the input data, easy to use as it requires a minimum number of user specified parameters for each step, and provides a summary in results of segmentation, registration, global signals, and motion-related artifacts (Esteban et al., 2019). Our software, FuNP, has the following advantages. First, FuNP contains both volume- and surface-based preprocessing pipelines. Using the surface-based pipeline, researchers can handle cortical and sub-cortical data better and more consistently with recent developments (Glasser et al., 2013, 2016a,b). Secondly, FuNP provides a fully automated preprocessing framework. Thirdly, FuNP is user-friendly owing to its graphical interface, which is intuitive and easy to manipulate. Fourthly, we designed our software so that the pipeline could be applied to fMRI data without field map data. This might be important because, in old neuroimaging studies, researchers often did not collect field map data. In such cases, modern researchers cannot use up-to-date preprocessing pipelines that require field map data. The reliability of FuNP was assessed by constructing RSNs using rs-fMRI data from the HCP database (Van Essen et al., 2013). Both the volume- and surface-based brain networks were well-defined and were consistent with pre-defined brain networks (Figure 6, 7). In addition to RSNs, the IQMs of temporal characteristics and artifacts were calculated to assess the quality of the preprocessed data. We found that FuNP outperformed FSL and fMRIPrep in terms of the IQMs (Figure 8). These results indicate that the developed preprocessing pipelines for T1-weighted structural MRI and fMRI data are of high-quality and reliable. Our software can be used as robust and easy-to-use neuroimaging data preprocessing framework.

There are several options to choose from to perform a given preprocessing step. Following statements are the justifications of the choices we made for each preprocessing step. Some choices (e.g., skull stripping) could be considered as optimal (Iglesias et al., 2011; Puccio et al., 2016), still, some could be suboptimal due to on-going controversies (e.g., nuisance removal) (Ciric et al., 2017). To remove the non-brain tissues, we selected “3dSkullStrip” function in AFNI rather than “bet” function in FSL, “antsBrainExtraction” function in ANTs, and “HWA” function in FreeSurfer. Previous studies reported that the function in AFNI outperformed equivalent functions in FSL and FreeSurfer for non-brain tissue removal (Iglesias et al., 2011; Puccio et al., 2016). A previous study reported ANTs showed better skull stripping results than other conventional approaches by visual inspection suggesting that our choice might be suboptimal (Esteban et al., 2019). For the step of magnetic field inhomogeneity correction, we chose “3dUnifize” function in AFNI out of coincidence. There are alternatives of “N4BiasFieldCorrection” function in ANTs and “fast” function in FSL. When performing registration, we chose “flirt” function in FSL. One study reported that neuroimaging registration could be better performed using “antsRegistration” function in ANTs compared to FSL and SPM (Dadar et al., 2018). Thus, we built two versions of FuNP. The new version adopted “antsRegistration” function and is referred to as FuNP v.2.0. We decided to keep the old version, referred to as FuNP v.1.0, because “flirt” requires fewer computation resources (i.e., runs fasters) compared to ANTs. For fMRI data registration, the FuNP used the two-stage registration that aligns the fMRI data to the T1-weighted structural data and then subsequently onto the MNI standard space. However, a previous study demonstrated that registration of fMRI data using echo planar imaging template improved the statistical power and reduced variability across subjects compared to the two-stage registration approach (Calhoun et al., 2017). Thus, our strategy for fMRI data registration might be suboptimal. In the tissue segmentation step, “fast” function in FSL was adopted that showed good performance compared to other algorithms (Eggert et al., 2012; Kazemi and Noorizadeh, 2014; Valverde et al., 2015). For slice timing correction, we chose “slicetimer” function in FSL and there are alternatives of “3dTshift” function in AFNI and “spm_slice_timing” function in SPM. For head motion correction, “mcflirt” function in FSL was adopted. It was shown that there was no single package that outperformed others for head motion correction (Oakes et al., 2005). There are many approaches to remove the nuisance variables in fMRI data such as head motion, cardiac, respiratory, WM, and CSF, but there is no single approach that can eliminate the artifacts completely (Ciric et al., 2017). A previous study reported that there were trade-offs among different strategies for nuisance variables removal and thus users need to select appropriate strategies in the context of their scientific goals (Ciric et al., 2017). In FuNP, “fix” function in FSL, the state-of-the-art approach, was adopted to remove nuisance variables of head motion, WM, CSF, cardiac pulsations, and arterial and large vein-related contributions (Salimi-Khorshidi et al., 2014). This approach requires the pre-trained datasets to classify between the signal and noise components (Parkes et al., 2018). The FuNP uses the pre-trained datasets that were trained using different image acquisition settings provided by the FSL team^2^. The users of FuNP need to choose which pre-trained data best suits their data being processed. Thus, the users do not need to manually train their data but choose from one of the several choices. However, if the input data were scanned with a very different image acquisition setting compared to existing choices, then “fix” function of FSL might not work well.

We compared the computational performances among three different software packages. We found that FuNP outperformed other software packages in terms of running time. This computational efficiency might be practical beneficial for preprocessing large-scale data which are likely to become more pervasive. A previous study reported that the total processing speed for registration accelerated two to three times when graphics processing unit (GPU) was adopted (Luo et al., 2015). The processing speed of recon-all, which was used for surface-based T1-weighted MRI data preprocessing in FuNP, could be improved 10 to 150 times with the help of GPU based computations according to the FreeSurfer official website^6^. We plan to update FuNP with GPU capabilities in the future.

## Conclusion

In this study, we incorporated existing software packages of AFNI, FSL, FreeSurfer, and Workbench to build a preprocessing pipeline for T1-weighted structural MRI and fMRI data. The developed software, FuNP, provides a fully automated and user-friendly GUI volume- and surface-based preprocessing pipelines. The FuNP showed good performance in terms of temporal characteristics and artifacts removal. We believe our pipeline might help researchers who need MRI data preprocessing.

## Data Availability Statement

The HCP dataset analyzed for this study can be found in the Human Connectome Project (http://www.humanconnectome.org/) upon request (Van Essen et al., 2013). The local dataset analyzed for this study are not publicly available.

## Author Contributions

B-yP and HP wrote the manuscript. KB aided the experiments. HP was the guarantor of this work, and as such, had full access to all the data in the study and takes responsibility for the integrity of the data and the accuracy of the data analysis.

## Conflict of Interest Statement

The authors declare that the research was conducted in the absence of any commercial or financial relationships that could be construed as a potential conflict of interest.

## Footnotes

1 http://www.fil.ion.ucl.ac.uk/spm/

2 https://fsl.fmrib.ox.ac.uk/fsl/fslwiki/FIX/UserGuide

3 https://research.ics.aalto.fi/ica/icasso/

4 https://gitlab.com/by9433/funp

6 https://surfer.nmr.mgh.harvard.edu/fswiki/CUDADevelopersGuide
