# Supplementary material for: FuNP (Fusion of Neuroimaging Preprocessing) Pipelines: A Fully Automated Preprocessing Software for Functional Magnetic Resonance Imaging
Source: Front Neuroinform. 2019 Feb 11;13:5. doi: 10.3389/fninf.2019.00005 (PMC6378808; doi:10.3389/fninf.2019.00005)
Supplement: Supplementary file 1 [file Data_Sheet_1.docx]

Supplementary Material

FuNP (Fusion of Neuroimaging Preprocessing) pipelines: A fully automated preprocessing software for functional magnetic resonance imaging

Bo-yong Park^1,2^, Kyoungseob Byeon^1,2^, Hyunjin Park^2,3*^

*** Correspondence:** Hyunjin Park: hyunjinp@skku.edu

# Supplementary Figures


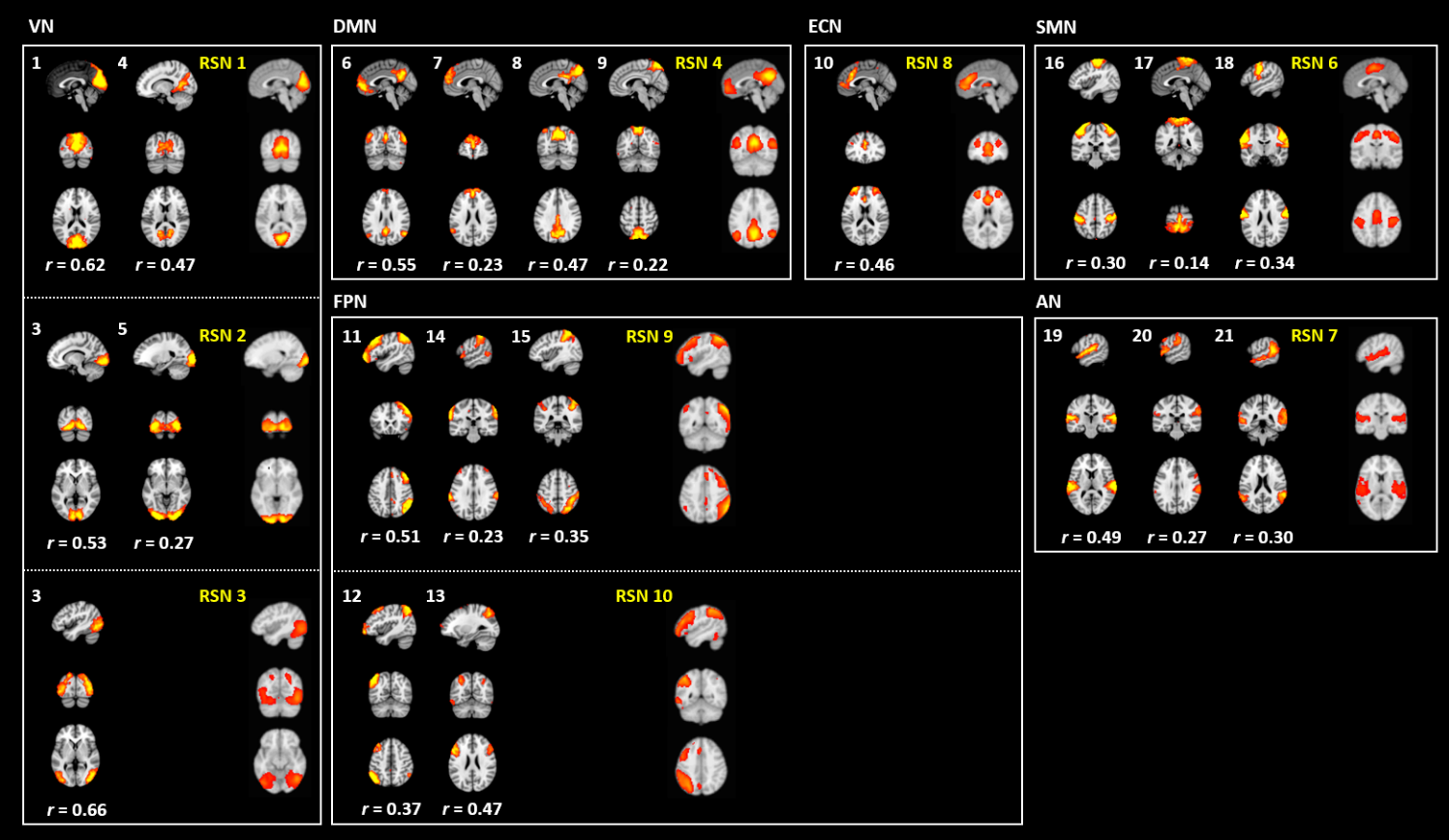
**Supplementary Figure 1.** Generated VICs using the local data (labeled in a white font) along with pre-defined RSNs (labeled in a yellow font) (Smith et al., 2009). The cross-correlation values of the spatial maps between the generated VICs and RSNs are presented.


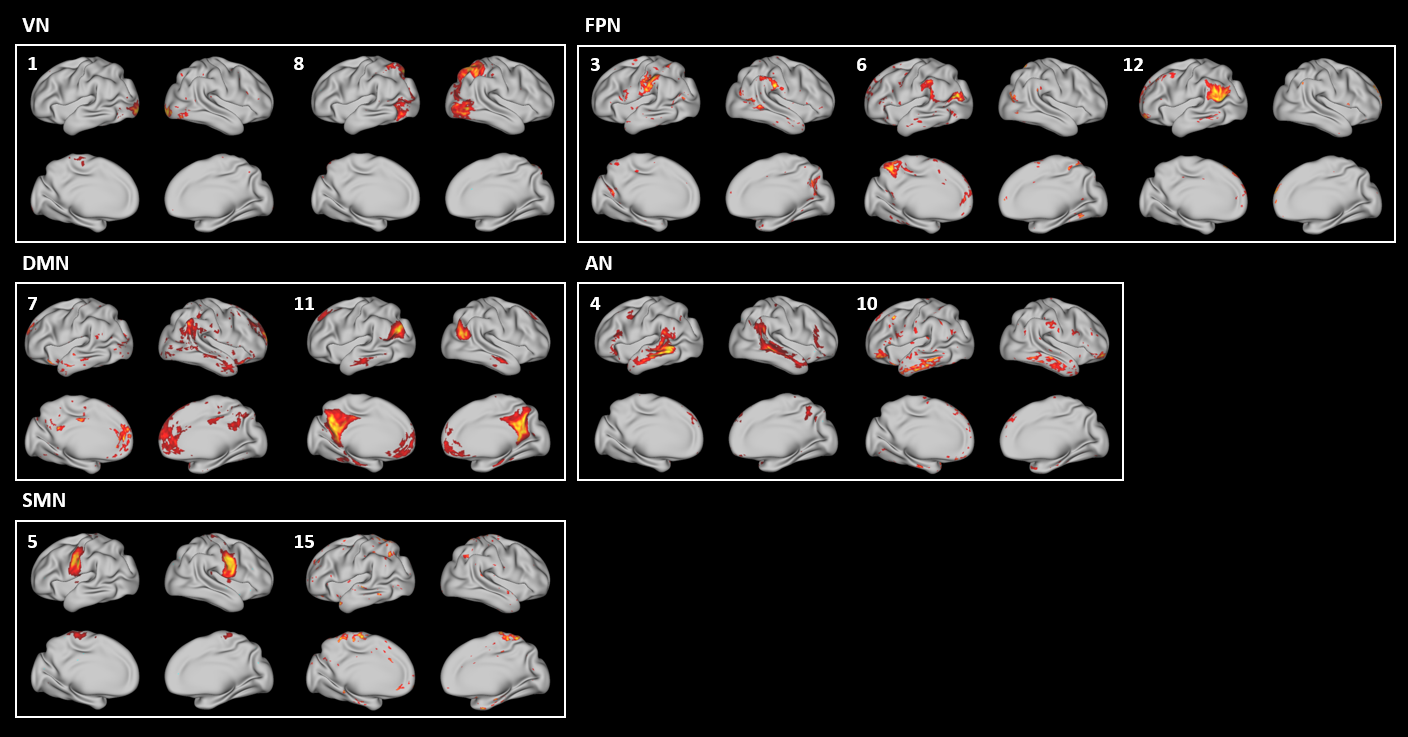
**Supplementary Figure 2.** Generated SICs using the local data matched with known RSNs.
